# The implementation of mentalization-based treatment for adolescents: a case study from an organizational, team and therapist perspective

**DOI:** 10.1186/1752-4458-6-10

**Published:** 2012-07-20

**Authors:** Joost Hutsebaut, Dawn L Bales, Jan JV Busschbach, Roel Verheul

**Affiliations:** 1 Viersprong Institute for Studies on Personality Disorders (VISPD), Halsteren, The Netherlands; 2 Department of Medical Psychology and Psychotherapy, Erasmus Medical Centre, Rotterdam, The Netherlands; 3 Department of Clinical Psychology, University of Amsterdam, Amsterdam, The Netherlands; 4 PO Box 7, 4660, AA, Halsteren, The Netherlands

**Keywords:** Implementation, Treatment integrity, Personality disorders, Adolescents, Mentalization-Based Treatment

## Abstract

**Background:**

Reports on problems encountered in the implementation of complex interventions are scarce in psychotherapy literature. This is remarkable given the inherent difficulties of such enterprises and the associated safety risks for patients involved.

**Case description:**

A case study of the problematic implementation process of Mentalization- Based Treatment for Adolescents (MBT-A), a new therapy for 14 to 18 year old youngsters with severe personality disorders, is presented. The implementation process is described and analyzed at an organizational, team and therapist level.

**Discussion and evaluation:**

Our analysis shows that problems at all three levels contributed and interacted to make the implementation cumbersome and hazardous.

**Conclusion:**

The implementation of complex psychotherapeutic programs for difficult patients could benefit from a structured attention to processes at multiple levels. We therefore propose a new comprehensive heuristic model of treatment integrity. This new model includes organisational, team and therapist adherence to the treatment model as necessary components of treatment integrity in the implementation of complex interventions. The application of this new model of treatment integrity potentially increases the chance of successful implementations and reduces safety risks for first patients enrolling in a new program.

## Background

The last two decades have yielded new and promising interventions for the treatment of borderline personality disorder (BPD). For example, several studies support the effectiveness of various psychosocial interventions for BPD in adults, including Mentalization-Based Treatment (MBT) [1], Dialectical Behaviour Therapy (DBT) [2], Schema-Focused Therapy (SFT) [3], Transference-Focused Psychotherapy (TFP) [4], Systems Training for Emotional Predictability and Problem Solving (STEPPS) [5] and Cognitive Behaviour Therapy (CBT) [6]. These results have typically been obtained under optimal (experimental) conditions, including extensive supervision, adherence monitoring, and above average organizational support. It is less clear how these evidence-based programs are actually implemented in regular practice. Given the many challenges associated with treating BPD patients and the complexity of these interventions, this issue might be particularly relevant to this patient group. Therefore, it is not only important to report about what works, but also to share experiences on how to implement these promising interventions. However, despite its obvious relevance, reports of (problems in) the dissemination of complex psychosocial interventions seem almost absent in the psychotherapy literature. In fact, we couldn’t find a single article describing implementation failures of a psychotherapy treatment program. It is unlikely that this absence of reports reflects actual absence of any implementation failures. Rather, we believe that problems are underreported and opportunities to learn from previously encountered problems are missed [7]. In other branches, such as the airline industry, reporting about problems and the lessons learned has been a successful strategy to increase safety [8,9]. In this article, we aim to introduce this strategy in the psychotherapy literature.

For that purpose, we will describe a case study of a problematic implementation of Mentalization- Based Treatment for Adolescents (MBT-A), i.e. a new treatment program for 14 to 18 year old youngsters with severe personality disorders, at de Viersprong, Netherlands institute for personality disorders. This case study revealed an intriguingly ambivalent result, i.e. patient outcomes were favourable in terms of symptom reduction and improvement of personality functioning and quality of life [10], while the program had to deal with numerous unexpected difficulties and threats for patient safety, including high staff turnover, temporary curtailment of the program, high level of patient and parent dissatisfaction, safety risks for patients and staff, and negative publicity. The analysis described in this article and the lessons learned from it, have stimulated and underpinned a new format for the program. The strongly adapted program now runs much smoother, while the favourable outcomes seem at least maintained, if not further improved.

As far as we know, this manuscript is the first published report on a failed implementation of a psychotherapy program. The problems described in this case are not likely to be specific to the implemented treatment model or specific setting, but instead might include various commonly encountered problems and thus are likely relevant for other treatment models and settings as well. Below, we will first introduce the case and describe the precursors to the implementation problems. Second, we will systematically analyze the encountered problems at an organizational, team and therapist level, respectively. Third, this analysis is used to reformulate the concept of treatment integrity in a way that could be useful to understand successes and failures in the dissemination of evidence based treatment models. This model might help to increase the chance of successful implementation of treatment programs and reduce safety risks for involved patients, staff and organization^a^.

### Case description

#### Problem definition

De Viersprong has approximately 40 years of experience in treating adolescents with personality problems in a long term inpatient setting. Traditionally, mildly to moderately disturbed adolescents entered this intensive and supportive treatment program and typically showed large improvements with effect sizes in the range between 1.0 and 1.5. Due to recent major organizational changes in mental health services in the Netherlands, including (a) the transition from supply- to demand-focused health care, (b) the differentiation between first (local), second (regional) and third (national) echelon mental health care, and (c) the introduction of stepped care as the basic principle in assignment to each of these echelons, de Viersprong – as a highly specialised, third echelon organization – started to attract a new population of more severely disordered adolescents. The inclusion of these more severely disordered adolescents posed new challenges to the therapists and organization. In particular, they were more sensitive to crisis, and displayed a wider range of externalizing problems. The prevailing treatment model – even with major adaptations – failed to adapt to the needs of these more severely disordered patients, leading to a sharp increase of dropout rates to almost half of the patients. It was obvious and recognized that the old program required substantial reorganization to be able to face the challenges this new patient group presented. In the process of reorganizing, part of the treatment program in the existing clinic was substituted for a new treatment program, i.e. MBT-A.

#### Choosing a new treatment model

The challenge was to design a treatment program that would be able to deal with the problems of severe borderline adolescents, often including behavioural problems, substance abuse, extreme self-injurious behaviour, extreme sensitivity to crisis, absenteeism, and severe family conflicts. At that time, our intention was to keep the inpatient setting and solve these problems by choosing a method aimed at dealing with the borderline symptoms. However, no randomized controlled studies on personality disorders (PD) in adolescents had been published nor were there guidelines on how to treat these adolescents. Eventually, MBT was chosen as the theoretical and methodological base for the new program. MBT is a psychodynamically oriented treatment program developed by Bateman and Fonagy [11,12] for adults with (severe) BPD. MBT was chosen for various reasons. First, MBT is one of the evidence-based treatment programs for adults with BPD. Second, MBT uses few exclusion criteria and in fact has been proven to be especially effective for very severely disturbed BPD patients [1,11,13]. Third, the model had not yet been applied to adolescents, but a similar approach had been described by Bleiberg [14] in Boston. Finally, and of great importance in this case, the adult MBT-program had been implemented successfully at de Viersprong before [15].

#### Preparing the implementation

About nine months passed between the choice for MBT and the actual implementation of the new program. During this period, a manual was written with some adaptations of the model to make it suitable for adolescents within an inpatient setting. This mainly included the addition of school and family therapy to the program and supporting developmental tasks, like structuring free time^b^. Furthermore, the team attended several congresses and presentations about MBT and discussed the manual and other relevant literature. Finally, the team was trained by experienced MBT trainers and the manual was revised and supplemented by the supervisors and team.

#### The start

Although the new therapy was hardly announced in professional journals or other media, many patients were admitted and almost immediately a waiting list developed. During the information sessions before the start of the new program, patients and their families reported that they had been waiting for a new specialized therapy and expressed high expectations. This apparent demand strengthened the belief that the clinical team had made the right choice in adapting their inpatient therapy into a specialized inpatient treatment for severe and/or resistant BPD in adolescents. All these factors further increased the already motivated and enthousiastic team spirit at the start of the new program in March 2008. However, it turned out that this high morale was quickly put to the test.

#### Signs of a failing implementation

It soon became clear that the implementation did not develop as expected. Signs were twofold and came from staff as well as from patients. Several staff members, particularly nurses, became overwhelmed and overburdened by the severity of the pathology they had to deal with combined with the ambiguity of their new tasks and role and uncertainty about MBT interventions. This left many nurses feeling powerless, resulting in a loss of authority and an increase of conflicts with patients. The resulting loss of morale and decreasing job satisfaction contributed to conflicts in the team and a burn out among several staff members. This interacted with increasing turmoil among the patient group, who also had to deal with major changes, including a new therapy schedule, a new therapeutic approach, and different rules. A vicious circle developed, with increasingly frustrated and overburdened staff and the youngsters feeling increasingly misunderstood, neglected and angry. All this resulted in an increase in acting out behaviour, more crossings of behavioural boundaries and a general grim and brutal atmosphere. Several nurses took sick leave due to stress and exhaustion, leaving the program understaffed, increasing the work load for the remaining staff. It became impossible to run daily therapy program five days a week. The program had to be limited to initially two and later three days a week. Parents were confronted with their children being at home most of the week, while they counted on them only being home in the weekend. Parents’ dissatisfaction escalated in an information meeting with the board of the management of de Viersprong, leading them to inform the National Health Care Inspection, several other organizations, the press and patients’ sites on the web. With all the negative press and pressure from patients and parents, the implementation problems reached their climax.

#### Immediate intervention and long term analysis

At that time, the board of management of de Viersprong intervened, although it would take some time before the measures would have some effect. The most important interventions were a stop in patient admissions and the quick recruitment of additional personnel creating an ‘overstaffing’ of the team. Overstaffing was necessary, to bring the staff at operational strength given the many sick leaves. Bringing the staff back to its intended operational strength restored the balance staff/patients and helped to regain a sense of control over the acting out behaviour of patients. Furthermore, the frequency of the supervision by experienced MBT therapists from within the institution was substantially increased. More time was scheduled to train new personnel and to discuss problematic team processes during intervision.

Looking back, the implementation turned out to be almost catastrophic, given the actual risks for patients, staff, and institution. It is therefore remarkable that dropout rates displayed a large improvement (less than 15 %) over the preceding treatment program (almost 50 %) and patient outcomes were actually not poor at all: symptom level and personality dysfunctioning decreased significantly with effect sizes ranging from medium to large [10]. Nevertheless, the beneficial effects of this new treatment should be weighed against the problems and costs associated with its implementation. These costs were considerable. First, patients and families experienced inconsistencies and unreliability, leaving many of them disillusioned. Second, staff had been confronted with much turmoil and crises, resulting in a high rate of illness absence during the first six months after the implementation. In the end, more than 75 % of staff members left the program as a direct or indirect consequence of the turbulence caused by the implementation problems. Third, the new program had caused some reputation damage to the institution and a considerable amount of budget had been reallocated from established programs to the new program. Finally, the crisis had created major operational risks within a relatively small organization.

Given these high costs, risks and burdens, it was considered very important to analyze the encountered problems thoroughly. During and after the efforts of regaining control at the ward, many meetings were organised and several reports were written in an effort to understand ‘what went wrong’. From these efforts, it became clear that there was no ‘magic bullet’ which could be pinpointed as responsible for all encountered problems. In the next paragraph, we will discuss in more detail the several interacting factors leading to the implementation failure.

## Discussion of problems at three levels

As in many circumstances of medical failure, it became clear that the failure could only be understood from a complex of interacting problems at different levels. Heuristically, we choose to differentiate between factors at the organizational, team and therapist level that contributed to the failed implementation. This analysis is completed with a discussion of factors related to the specific choice of method (inpatient MBT for adolescents). Finally, we discuss from a mentalizing perspective how all these levels have interacted to create a cascade-effect leading to the major problems as mentioned before.

### Organizational factors

The following organizational factors have contributed to the implementation problems: organizational structures, institutional culture and support, lack of structures to support change management, and staffing, logistics, and budget planning.

· Organizational structures

First, as in many institutions, the organization was divided in an adult and youth ward. Both were organizationally separated and managed by different managers. Expertise on the treatment of BPD with MBT was available in the adult ward and through these organizational barriers less easily accessible in the youth ward. Second, the organization had recently gone through a reorganization: management responsibilities were decentralized toward lower hierarchical levels in the organization. As a consequence, most of the managerial setup of the new program was assigned to psychotherapists, lacking relevant managerial experience and expertise. As such, the reorganization contributed to an insufficiently prepared project.

· Institutional culture and support

The organization was in transition from a traditional therapeutic community with relatively little interest in research and evidence based thinking towards a modern, science-oriented organization, resembling the processes described by Chiesa and Healy [16]. At the time of implementation, this transition was still accompanied by growing pain, expressing itself most tangibly in heated discussions about the future role of various traditions such as the centrality of ‘milieu therapy’ as one of the cornerstones of the institution. New programs like MBT and new patient groups like adolescents with severe externalizing problems did not fit in the institution’s traditional treatment philosophy. As the new program was considered to represent the reform within the context of ongoing debate, the new program lacked support from several key persons within the institution and as a consequence was cut off from input of experienced therapists in the organization. Moreover, any discussion or critical remark concerning the new program seemed to reflect this fundamental debate and therefore failed to be included in the implementation process in a constructive way. As a result, the program lacked broad support within the institution, leading to an accumulation of critical remarks after the problems arose. Boundary crossings by the adolescents were interpreted as a justification of this opinion. The team became isolated within the institution.

· Lack of structures to support change management

The existing program at the ward had been more or less unchanged for more than 40 years. Several staff members worked for more than 20 years at the ward and were strongly attached to the old program. The existing program was rooted in a Therapeutic Community tradition which had been for decades the core landmark of the institution. The amount and degree of change for all involved parties implied by treating a different population with a different method demanding a different team culture had been largely underestimated. No specific structures to support these changes had been established. As a result, many implementation issues had to be dealt with while already running the program and problems had to be solved ‘on the spot’. Further on, potential risks and pitfalls involved in these major changes had not been sufficiently identified at forehand, based upon an analysis of the existing situation and the desired changes.

· Staffing, logistics and budget planning

Due to a lack of experience and a hasty and premature start of the new program, the implementation plan showed major shortcomings. Among the shortcomings were: insufficient staffing due to two vacant positions, insufficient logistics and facilities as the building had to serve two different programs instead of one unified program, a selection of staff members with insufficient competencies to deal with the complex needs of the new group of patients, and insufficient budget planning and evaluation. The new program not only required budget for training and supervision, but also an increase in personnel, and a financial buffer for unexpected expenses. Once the problems arose and absent staff had to be replaced in order to be able to continue the program, financial expenses exceeded the planned budget, raising even more the organizational pressure on staff.

### Team factors

The following team factors have contributed to the implementation problems: team problems prior to the implementation, resistance toward change, lack of clear leadership, communication difficulties, and lack of clear supervisory structures.

· Team problems prior to the implementation

A major reason for changing treatment methods was the experienced shortcomings of the existing model to deal with the new population of crisis-sensitive adolescents. In the absence of a clear method, this had lead to differences in opinions within the old team about how to deal with crisis. Thus, even before the new program started, there was an imminent split in the team, mainly between psychotherapists and nurses. This split was partly due to the nature of the patients in treatment, and their tendency to split their projections on staff members. The psychotherapists were often idealized by the patients, whereas the psychosocial nurses (whom were ´available´ 24 hours a day) frequently had to deal with the negative projections partially due to their pedagogical role. This strengthened the wish to quickly implement the new program as a possible solution for these differences in opinions. However, as the split wasn’t well enough understood, it re-emerged quickly when the arousal increased at the ward due to all unexpected difficulties with the implementation. Old team dynamics kept influencing the new way of working.

· Resistance toward change

The new program required changes within the team at different levels. The traditional therapeutic community was characterized by much democracy without a clear demarcation of leadership. The new model required a new hierarchical order with psychotherapists being in lead as the primary clinicians. Further on, the members of the new and old team were identical. Many therapists found it hard to give up their ´old´ theoretical model and routine way of treatment. Despite huge efforts to use the new concepts and philosophy, a subtle mixture of old and new ways of thinking and handling was inevitable, leading to minor and larger inconsistencies in applying the MBT model. With the increase of stress at the ward came an increase in inconsistencies in treatment and an increase in patient (and staff) crisis.

· Lack of clear leadership

Partly due to the old ‘democratic’ culture of the prior therapeutic community, the team lacked clear and broadly supported leadership in this moment of change. This meant that the team was not only experimenting with a new therapy, but at the same time experimenting with its own management.

· Communication problems

The team was large and it turned out - due to the (typically part time) working schedules - to be impossible for the whole team to attend intervision-supervision on a regular basis. These factors made it difficult to communicate relevant patient information well enough between the team members, Relevant patient information got ‘lost’ between shifts, leading to increasing arousal among patients whom felt ‘forgotten’ by the staff. Moreover, the inability to attend intervision also partially denied staff members the emotional support and learning opportunity in talking over the difficult cases and problematic situations encountered.

· Lack of clear supervisory structures

Supervision and training were offered, but due to the organizational barriers, from a distance. More generally, the treatment manual was experienced as too abstract, and the team lacked an experienced supervisor who could help translate theory into practice, guide and monitor interventions and help manage team processes from a mentalizing perspective. What was lacking, was supervision ‘on the spot’, highly needed given the lack of experience in the team and the challenging population.

### Therapist factors

The following therapist factors have contributed to the implementation problems: personnel selection, and lack of experience with the model.

· Personnel selection

The old team – selected and trained for the purpose of treating a different population of adolescents – was re-educated in the new model. There had been no explicit selection of personnel based upon their abilities to treat a more severe BPD-population, using a different method, focussing strongly on affective and relational issues. Soon after the start, some personnel felt less comfortable with the new demands that were put upon them by the new program.

· Lack of experience with the model

None of the therapists had previous experience with the new model. Although therapists were trained in the model, had read the books and manual, and received classic supervision by experienced trainers, they still felt insufficiently prepared to apply their new knowledge and skills to deal with everyday changing situations. Therapists from all disciplines experienced a lack of concrete supportive protocols to deal with frequent clinical problems like youngsters being absent from therapy or school, engaging in or threatening with self-injurious behaviour, staying in bed, insulting and provoking team members or peers, or refusing to obey general rules of the unit. This lead among several therapists to increased uncertainty about how to apply the model on a daily basis. Especially when problems increased, the team morale dropped as did the belief in the usefulness of the model to face the challenges met in their work with these patients. This further hindered the use of this new theoretical framework in a consistent way from the start, and to use the mentalizing method as a new cornerstone for team functioning and interactions.

### Factors related to the choice of inpatient MBT for adolescents as the new model

Finally, implementation problems could in part also been explained by the specific choice of model and setting. Three issues contributing to the problems at this level are the choice for MBT, the choice for an inpatient setting, and the necessary adaptations for adolescents. All three added to the complexity of the innovation.

· MBT

Compared to the existing program, the MBT-model required a totally different sort of stance and range of interventions, requiring different skills from therapists. MBT emphasizes the development of an attachment relationship with patients, staying mentally close even in times of crisis and adopt a not-knowing stance. It requires a level of transparency from therapists unlike other models and aims at focussing on affective issues within the therapist-patient relationship. All these core characteristics of MBT required to some degree different personality characteristics from staff members.

· Inpatient setting

The amount and intensity of contact among patients within an inpatient setting is much greater than in an outpatient setting, leading to (hyper)activation of the attachment system and correspondingly higher levels of stress at the ward [17]. Also, within an inpatient setting, the team size is larger, making it more difficult to offer a coherent and consistent approach, which in turn added to a lack of consistency in communication and thus ‘unreliability’ in the communication as experienced by the youngsters. Acting out increased under such circumstances, having a large emotional impact on nurses because they often had to deal with boundary crossings or parasuicidal actions.

· Adolescent population

MBT had originally been developed for adult BPD patients. Therefore, it required adaptation to meet the specific needs of adolescents. For example, pedagocial limit setting turned out to be an important issue which was insufficiently covered by the manual and trained in the classic training. Further on, some characteristics of adolescents seemed to make (especially) group therapy more complex. Their decreased capacity to mentalize contributed to their difficulties to differentiate from peers, leading to high levels of arousal within group therapy and strong loyalty towards each other. This sometimes led them to cover up each other’s boundary crossings and to try managing each other’s complex problems without discussing them with adult staff members.

In sum, we believe an important contributing factor to the implementation problems, was the amount of innovation involved. Treating adolescents for their underlying personality pathology was new at that time; adapting MBT to an inpatient setting was new as was treating adolescents with MBT. There was a lack of (published) experiences on the ‘shoulds’ and ‘shouldn’ts’ in adapting MBT to an inpatient adolescent BPD population. The high level of innovation in itself created major challenges that should have been addressed more extensively prior to the start in the implementation plan.

### A cascade of negative interactions from a mentalizing point of view

It should be clear that all these factors interacted to create a snowballing effect, leading to increasing levels of arousal at the ward and demoralization and anxiety among staff. The huge amount of changes implied by the new program combined with the lack of experience with the new model and the insufficient guided implementation increased feelings of uncertainty and incompetence among staff. In a team that was lacking clear leadership and clear supervisory structures, this lead to inconsistencies in the approach of youngsters. Especially staff members whom felt less comfortable with the new demands from the model, felt uncertain and insecure in an unstable and already conflicted team. Their mentalizing abilities reduced, leaving them more vulnerable to act out towards youngsters, like withdrawing from contact. In turn, the experienced unreliability increased anxiety, activated the attachment system, reduced mentalizing capacity and increased acting out among youngsters (whom are already vulnerable to lose their mentalizing abilities due to developmental changes and their peer bonding in an inpatient setting), often using the more uncertain staff members to project their anxieties and anger upon. Again, this further reduced the mentalizing abilities of these staff members, further increased by the lacking competencies of the team to adopt a mentalizing stance towards team interactions, leading to insufficient support for staff members under stress. In turn, these processes increased splitting within the team, making it even more difficult to maintain a mentalizing stance towards each other. The resulting splitting further contributed to inconsistencies in the approach of youngsters, which again further increased their arousal. Under the influence of the arising problems, previous scepticism from the broader context turned into severe criticism, leading to a defensive withdrawal of the team. The relation between the team and the rest of the organisation got infected by increasing mutual distrust and resulting problematic communication. This not only denied the team from further emotional and supervisory support but also hindered regaining a mentalizing perspective on team functioning. This contributed to the split within the team, the lack of experienced emotional support and the increasing stress. As a result, a cascade of negative interactions and effects finally lead to exhaustion and complete demoralization of several team members.

### Solving the implementation problems

As has been described earlier, the implementation crisis warranted immediate action from board of management of de Viersprong, including a temporary patient stop, the addition of specific MBT expertise to the program, and the recruitment of new personnel. These interventions helped to regain basic control, diminish the turbulence, and improve the quality of the program. However, our extensive analysis of the implementation problems inevitably led to a radical reorganisation of the program in line with the resulting conclusions of this analysis. This reorganisation has been designed along the following lines:

1. *Reducing the complexity of the program*. A major contributing factor turned out to be the high level of innovation, complexity and intensity implied in treating BPD adolescents with MBT for longer periods of time in an inpatient setting. Therefore the inpatient setting was replaced by an outpatient setting. It was assumed that this would reduce the burden of staff members, increasing their ability to maintain a mentalizing stance, individually and as a team. The format of the intensive outpatient version of MBT for adults was used, as has been described in detail and studied by Bateman and Fonagy [18]. Thereby, the program was based upon an ‘evidence based format’ and improved opportunities to work in smaller teams and enhance consistency. Furthermore, the age range was restricted from 14–18 to 16–18 years. It was assumed that less developmental heterogeneity would also reduce complexity.

2. *Embedding the new program within existing expertise*. As has been described, the lack of organizational embedding led to missed opportunities to use existing expertise from within the institution. Therefore, the new program was organizationally embedded within a newly formed MBT unit that integrated the formerly existing adolescent and adult MBT programs. This enabled us to integrate MBT and adolescent expertise in the organization.

3. *Designing a new implementation plan for the new program*. In contrast to the cumbersome start of the first version of the adolescent MBT program, the new version was started ‘*de novo’*. An implementation plan was designed addressing the various issues mentioned before in this article, including referral process, personnel recruitment, logistics, facilities, and so on. The implementation plan was discussed at all organizational levels, ensuring enough support within the institution.

4. *Embedding the program within the development of a quality system*. One of the major issues was the lack of familiarity with the model on a daily base and the associated difficulties to maintain a reflective, mentalizing stance within the team interactions. This led to reduced therapist adherence to the model and increased interfering team processes. To increase adherence to the working mechanisms of the model and to decrease potentially damaging processes, a quality monitoring system is now being developed. For example, each treatment program has a supervisor who is not a therapist working in that team. The supervisor is an experienced MBT therapist needing several skills: he of she has to be able to measure and reflect on MBT interventions and adherence, to enhance therapist’s mentalizing stance, and to signal and manage destructive team processes like unnoticed or unrevealed splits.

5. *Developing an organizational manual.* The organizational manual is a manual on management and service organization in which the managerial aspects of designing and maintaining a MBT service are described. Management focus is on organizing and facilitating the clinical processes as written up in the treatment guide and monitored within the quality system and is thus an important part of the quality system.

The program now runs in this new format. The implementation has been remarkably smoother. Results are being monitored. Although we do not have research-based results yet, the first clinical impressions support the effectiveness of the program in line with our previous findings of the old MBT program for adolescents.

## Conclusion

In this paper, we have argued that the escalating implementation problems in this case study could best be understood from the interaction of three levels of application: organization, team and therapist. At an organizational level, the organizational barriers and recent reorganisation, lack of support within the institution due to the institutional culture, lack of structures to support change management and the shortcomings in the implementation plan, were considered to be the dominant problem factors. At team level, the team problems prior to implementation, the resistance toward change, lack of clear leadership, communication problems and lack of supervisory structures were important determinants. At therapist level, the lack of selection of personnel and the lack of experience with the new model contributed to the implementation problems. These problems were further magnified due to the level of innovation implied in adapting MBT for an inpatient adolescent population. Together, these factors led to increasing impotence and frustration in staff, interacting with increasing levels of arousal and distrust in the patient group related to experienced unreliability. Increased patient turmoil, staff exhaustion and safety risks for patients, staff and the institution ended up resulting in ending the inpatient treatment program.

In our view, the analysis of this case study might have implications for the conceptualization of treatment integrity to explain successful implementation of evidence-based programs. In psychotherapy outcome research, treatment integrity refers to the extent to which the intervention was implemented as intended [19]. It usually includes three determining components: treatment adherence, therapist competence and treatment differentiation [20]. Adherence refers to the degree of utilization of specified procedures by the therapist. Competence refers to the level of skill and judgment shown by the therapist in delivering the treatment. Differentiation refers to whether treatments under investigation differ from each other along critical dimensions. In short, treatment integrity classically refers to ‘good therapists’, i.e. therapists having the skills (competence) to perform the procedures as prescribed by the treatment manual (adherence). Based on our analysis, we propose to extend the concept to include also adherence, competence and differentiation at the level of teams and organisations. Especially in cases of the implementation of complex, innovative interventions for highly challenging patient groups, the reduction of the concept of treatment integrity to therapist adherence and competence might severely underestimate the influence of organisational and team issues in acquiring treatment integrity for such complex programs. In order to encompass these aspects of treatment integrity, we propose a 3x3 model, including three components of treatment integrity (i.e. *1* adherence, *2* competence, *3* differentiation) at three levels of application (i.e., *1* organisational (macro) level, *2* team (meso) level, and *3* therapist (micro) level). Examples of these components of treatment integrity, applied to the model of Mentalization-Based Treatment, are provided in Table1. 

**Table 1:** Multilevel model of treatment integrity including three components of treatment integrity at three levels of application

**Component of treatment integrity Level of application**	**Adherence**	**Competence**	**Differentiation**
**Therapist-micro level**	E.g. Not knowing, mentalizing therapeutic stance , main focus is on enhancing mentalizing within the context of attachment relationship, continuously adapting interventions according to mentalizing capacities of the patient, …	E.g. Motivated, professional attitude, feeling responsible, flexible, creative, open minded; being able to deal with crises, in situations under high arousal being able to keep mentalizing stance; team player, being reflective in contact with colleagues about their own mental states,…	E.g. avoiding focus on behavioural expressions and skills; avoid classic use of transference to promote personality change; no use of suicide contracts,…
**Team-meso level**	E.g. Continuous efforts to deliver a consistent and coherent treatment and to provide continuity within the treatment; stimulating mentalizing stance within team interactions to help in keeping a mentalizing environment,…	E.g. creating a large enough team to provide consistency and continuity even during holidays or sick leave of team members; well-balanced team with clear roles; at least one team, member should have authority and personality to create and maintain holding environment for the team; organizing a supervisory structure to increase MBT knowledge and competence of all team members and to ensure adequate managing of team processes.	E.g. intervision, supervision, group reflection and consultation, all stimulating mentalizing and aimed at enhancing adherence and competence
**Organization-macro level**	E.g. commitment to fully implement MBT; creating support within the whole organization for the new program; maintaining regular contact with important stakeholders; designing a detailed implementation plan,,…	E.g. sufficient budget to be able to implement the program; capacity of management to remain calm even if crises occur at patient level	E.g. implementation of a quality assurance system, including continuous monitoring feedback

*Adherence* refers to the degree to which processes and the procedures that help to optimize the working mechanisms of the model are utilized, i.e. (in case of MBT) what should therapists, teams and organisations do to enhance mentalizing among patients? At therapist level, adherence refers to the basic attitude and interventions that are described in the treatment manual. For example, referring to MBT, therapist adherence refers to the interventions described to enhance mentalizing in patients from a ‘not-knowing’ mentalizing stance. At team level, adherence refers to necessary team processes enabling the working of the model. For example, MBT requires coherence, consistency and continuity in team work. Inconsistencies (and possible splitting) in team functioning will cause confusion, destabilization, and subsequently an increase in crisis and other destructive behaviour. A consistent approach on the other hand creates reliability and safeness, while continuity helps to reconnect the fragmented experiential world of BPD patients. At organisational level, adherence refers to the managerial and organisational procedures that ensure the necessary conditions to implement and maintain the treatment program successfully. For example, to implement MBT, it is necessary that the organisation creates support for the new program within the own institution as the new, ‘difficult’, patients might interfere with the working of other wards. Another example of organisational adherence is the designing of a sufficiently detailed implementation plan, in which the innovative character of the program needs to be thought and worked through.

*Competence* refers to the level of skill and judgement shown in the delivering of the treatment, i.e. what basic qualities and skills should therapists, teams and organization should have in order to be able to perform the procedures as outlined in the ‘adherence’ section. At therapist level, competence for MBT refers to the basic qualities of therapists to work with the most complex BPD patients, including their ability to keep a reflective, mentalizing stance under high pressure. At team level, competence in MBT refers to the qualities a team should have to be able to maintain a consistent approach. This includes a well balanced team with clear roles and leadership qualities among at least one team member. At organisational level, finally, competence refers to the organisational and managerial qualities necessary to provide the organisational conditions for delivering this particular treatment. An evident example is sufficient budget to run the new program as it is intended. Another example, related to the complexity of the MBT population, is the ability of (managerial) people in charge to stay calm and intervene constructively after major crisis among the patients (for example, after a suicide).

*Differentiation* refers to what makes this program ‘unique’. As a concept, there is some overlap with the concept of adherence. At therapist level, differentiation refers to the interventions specific for this model and to the ‘forbidden’ (i.e. ‘non-mentalizing’) interventions. For example, in contrast to Dialectical Behaviour Therapy, MBT will focus much less on behavioural sequences and will instead focus on underlying mental states. At team level, differentiation should describe the specifics of the team functioning and communication within the particular model. For example, within MBT, communication among team members should be focused at helping each other to restore mentalizing. At organisation level, differentiation refers to the specific managerial issues and challenges for this model. For example, MBT might in the future encourage organisations to implement a quality control system, which requires organisational embedding and funding different from other treatment models for BPD.

We do not suggest this multilevel model of treatment integrity to be necessary to understand successes and failures in all treatment programs. However, the more complex a treatment model and the more complex the patient population, the more relevant it might be to use this more extended heuristic model of treatment integrity to have an overview of all procedures and qualities that should be provided in order to implement and maintain the treatment program successfully. For example, team adherence becomes a relevant issue when the treatment model requires an integrated (multidisciplinary) team to deliver a consistent treatment. Organisational adherence will be especially relevant when the new program is highly innovative for the organisation where it will be run. If this analysis is correct, this might also imply that the broadening of the concept of treatment integrity could have important implications for developers of complex treatment models. For example, a treatment manual might be a necessary, but insufficient tool to promote adherence in cases of complex (psychotherapeutic) interventions. Developers should also describe how the model should be implemented successfully at local settings within existing teams and existing organisations. This proposal is consistent to the approach taken by the developers of Multisystemic Therapy (MST) [21], who have described the organisational embedding in a separate managerial manual. We believe their approach could be inspirational for developers of other treatment models as well.

## Endnotes

^a^The WMO (Law Medical scientific research with human beings in the Netherlands) does not cover retrospective case studies like the one described in this report. The report does not describe a medical scientific experiment nor does it report on any additional action by patients in order to collect the data for this study. As a consequence no written informed consent was collected from the patients involved in the treatment described.

^b^As our aim is not the discussion of a treatment program for adolescents, but the illustration of a failed implementation, we limit ourselves for this purpose to this short description.

## Abbreviations

BPD : Borderline personality disorder; CBT : Cognitive behaviour therapy; DBT : Dialectical behaviour therapy; MBT : Mentalization-based treatment; MBT-A : Mentalization-based treatment for adolescents; MST : Multisystemic therapy; PD : Personality disorder; SFT : Schema-focused therapy; STEPPS : Systems training for emotional predictability and problem solving; TFP : Transference-focused psychotherapy.

## Competing interests

All authors are currently working for the organization. Three authors (JH, DB, RV) were directly involved in the implementation process of the treatment program. The organization is financing the manuscript.

## Authors’ contributions

JH was a involved in the MBT-A program and made the draft for the manuscript. DB was supervisor for the program, read the first draft and added several points of discussion. JB introduced the quality perspective in the article and edited the second draft. RV edited the last draft. All authors read and approved the final manuscript.

## References

[B1] Bateman A Fonagy P Eight-year follow-up of patients treated for borderline personality disorder Am J Psychiatry 2008 165 631 638 10.1176/appi.ajp.2007.07040636 18347003

[B2] Linehan MM Comtois KA Murray AM Brown MZ Gallop RJ Heard H Korslund KE Tutek DA Reynolds SK Lindenboim N Two-year randomized controlled trial and follow-up of dialectical behavior therapy vs therapy by experts for suicidal behaviors and borderline personality disorder Arch Gen Psychiatry 2006 63 757 766 10.1001/archpsyc.63.7.757 16818865

[B3] Giesen-Bloo J van Dyck R Spinhoven P van Tilburg W Dirksen C van Asselt T Kremers I Nadort M Arntz A Outpatient psychotherapy for borderline personality disorder: randomized trial of schema-focused therapy vs transference-focused psychotherapy Arch Gen Psychiatry 2006 63 649 658 10.1001/archpsyc.63.6.649 16754838

[B4] Clarkin JF Levy KN Lenzenweger MF Kernberg OF Evaluating three treatments for borderline personality disorder: a multiwave study Am J Psychiatry 2007 164 922 928 10.1176/appi.ajp.164.6.922 17541052

[B5] Blum N Pfohl B St.John D Stuart S McCormick B Allen J Arndt S Black DW Systems training for emotional predictability and problem solving (STEPPS) for outpatients with borderline personality disorder: a randomized controlled trial and 1-year follow-up Am J Psychiatry 2008 165 468 478 10.1176/appi.ajp.2007.07071079 18281407 PMC3608469

[B6] Davidson K Norrie J Tyrer P Gumley A Tata P Murray H Palmer S The effectiveness of cognitive behavior therapy for borderline personality disorder: results from the borderline personality disorder study of cognitive therapy (BOSCOT) trial J Personal Disord 2006 20 450 465 10.1521/pedi.2006.20.5.450 PMC1852259 17032158

[B7] Mahajan RP Critical incident reporting and learning Br J Anaesth 2010 105 1 69 75 10.1093/bja/aeq133 20551028

[B8] Hatcher S Risk management in mental health: applying lessons from commercial aviation Australasian Psychiatry 2012 18 1 4 6 20136528 10.3109/10398560903180125

[B9] Wilf-Miron R Lewenhoff I Benyamini Z Aviram A From aviation to medicine: applying concepts of aviation safety to risk management in ambulatory care Quality Safety Health Care 2003 12 35 39 10.1136/qhc.12.1.35 PMC1743670 12571343

[B10] Laurenssen EMP Hutsebaut J Feenstra DJ Bales DL Luyten P Busschbach JJV Verheul R Feasibility of Mentalization-based Treatment for adolescents with borderline symptoms: a pilot study Psychiatry Research Submitted 10.1037/a0033513 24059741

[B11] Bateman A Fonagy P Effectiveness of partial hospitalization in the treatment of borderline personality disorder: a randomized controlled trial Am J Psychiatry 1999 156 1563 1569 10518167 10.1176/ajp.156.10.1563

[B12] Bateman AW Fonagy P Psychotherapy for Borderline Personality Disorder: Mentalization-Based Treatment 2004 Oxford University Press, Oxford

[B13] Bales D Bateman AW Bateman AW, Fonagy P Partial Hospitalization Settings Handbook of Mentalizing in Mental Health Practice 2012 American Psychiatric Publishing Inc, Washington DC 197 225

[B14] Bleiberg E Treating Personality Disorders in children and Adolescents: A Relational Approach 2001 Guilford Press, New York

[B15] Bales D van Beek N Smits M Willemsen S Busschbach JJV Verheul R Andrea H Treatment outcome of 18-month, day hospital Mentalization-Based Treatment (MBT) in patients with severe borderline personality disorder in the Netherlands J Personal Disordin press 10.1521/pedi.2012.26.4.568 22867507

[B16] Chiesa M Healy K The struggle to establish a research culture in the psychotherapy hospital: reflections from the Cassel Hospital experience Bull Menn Clin 2009 73 157 175 10.1521/bumc.2009.73.3.157 19807221

[B17] Shaver PR Mikulincer M Attachment-related psychodynamics Attachment and Human Development 2002 4 243 257 10.1080/14616730210157484 12467506

[B18] Bateman A Fonagy P Randomized controlled trial of outpatient Mentalization-Based Treatment versus structured clinical management for Borderline personality disorder Am J Psychiatry 2009 2009 166 1355 1364 19833787 10.1176/appi.ajp.2009.09040539

[B19] Vermilyea BB Barlow DH O’Bren GT The importance of assessing treatment integrity: An example in the anxiety disorders J Psychopathol Behav Assess 1984 6 1 11

[B20] Perepletchikova F Kazdin AE Treatment integrity and therapeutic change: issues and research recommendations Clinical Psychology 2005 12 365 383

[B21] Henggeler SW Schoenwald SK Borduin CM Rowland MD Cunningham PB Multisystemic Therapy for Antisocial Children and Adolescents 2009 Guilford Press, New York

